# Lymphocyte Display: A Novel Antibody Selection Platform Based on T Cell Activation

**DOI:** 10.1371/journal.pone.0007174

**Published:** 2009-09-24

**Authors:** Vanesa Alonso-Camino, David Sánchez-Martín, Marta Compte, Laura Sanz, Luis Álvarez-Vallina

**Affiliations:** Molecular Immunology Unit, Hospital Universitario Puerta de Hierro, Madrid, Spain; University of Miami, United States of America

## Abstract

Since their onset, display technologies have proven useful for the selection of antibodies against a variety of targets; however, most of the antibodies selected with the currently available platforms need to be further modified for their use in humans, and are restricted to accessible antigens. Furthermore, these platforms are not well suited for *in vivo* selections. We present here a novel cell based antibody display platform, which takes advantage of the functional capabilities of T lymphocytes. The display of antibodies on the surface of T lymphocytes, as a part of a chimeric-immune receptor (CIR) mediating signaling, may ideally link the antigen-antibody interaction to a demonstrable change in T cell phenotype, due to subsequent expression of the early T cell activation marker CD69. In this proof-of-concept, an *in vitro* selection was carried out using a human T cell line lentiviral-transduced to express a tumor-specific CIR on the surface, against a human tumor cell line expressing the carcinoembryonic antigen. Based on an effective interaction between the CIR and the tumor antigen, we demonstrated that combining CIR-mediated activation with FACS sorting of CD69^+^ T cells, it is possible to isolate binders to tumor specific cell surface antigen, with an enrichment factor of at least 10^3^-fold after two rounds, resulting in a homogeneous population of T cells expressing tumor-specific CIRs.

## Introduction

The display of foreign polypeptides and proteins on the surface of viruses or cells provides an important tool for the engineering of biomolecules and the analysis of their interactions with binding partners [1]–[3]. The microbial surface display technologies for screening antibody libraries include phage, yeast and bacteria platforms [4]. Cell-free systems, as ribosomal display, are also available [4].

Although the above mentioned systems have been successfully used for the isolation of antibodies, concerns may rise about the lack of post-translational modifications and proper folding of selected proteins. In order to allow processing of the displayed antibody in a eukaryotic environment, screening systems based on viruses and mammalian cells have been described.

The potential of retroviral display for the generation and screening of eukaryotic expression libraries has been demonstrated for small peptides [5], [6] and antibodies [3], [7]. Remarkably, antibodies selected from retroviral libraries exhibit CDR sequences clearly different to those of antibodies selected from phage libraries [3]. We could hypothesize that these differences arise from the adaptation of molecular parameters, such as codon usage, polypeptide folding and resistance to inactivation by mammalian cell proteases. Although other mammalian cell surface display platforms have been developed [8]–[11], such methods, similar to phage and ribosome display, are enrichment techniques based purely on physical interactions and require multiple rounds of selection to be carried out. This is an important drawback; specially taking into account the limited number of antibodies that can be screened in a mammalian cell-based platform

In this report, we describe the design and testing of a mammalian cell surface display platform in T lymphocytes. The display of single chain antibodies (scFv) on the surface of T lymphocytes, as a part of a fusion protein mediating signaling, may ideally link the interaction of scFvs with their antigen to a demonstrable change in T cell phenotype, due to subsequent expression of activation molecules. Chimeric immune receptor (CIR) genes are composed of a recognition unit attached to the transmembrane and intracytoplasmic sequences of a signalling molecule derived either from the ζ chain of the TCR/CD3 complex or from the γ chain associated with some Fc receptors (FcR) [12]. CIRs enable us to target various types of effector cells toward any tumor-associated antigen (TAA) for which a suitable mAb exists [13]. These Ag-selective cell therapies have been designed to convert therapeutically important TAAs, expressed on the cell surface, into recruitment points of effector functions, and to address the goal of MHC- and exogenous cytokine-independent activation of effector cells, highly restricted to tumor areas. Upon encountering antigen, the interaction of the grafted CIR triggers effector functions and can mediate cytolysis of tumor cells [14]. The utility and effectiveness of the CIR approach have been demonstrated in a variety of animal models where tumor-specific CIRs drove the adoptive transferred autologous T lymphocytes to accumulate at the tumor site *in vivo* and prevented tumor growth [13], [14].

The platform presented in this paper allows a positive selection based on both the existence of an effective interaction, and the expression of the early T cell activation marker CD69. The surface level of CD69 can be used both as a “quality control” of antibody interaction, and as a sorting parameter. Using a human T cell line, gene-modified to express a tumor-specific CIR on their surface, and a human tumor cell line we demonstrated that combining CIR-mediated activation with FACS sorting of CD69^+^ T cells, it is possible to isolate binders to tumor specific cell surface antigen, with an enrichment factor of 10^3^ **-**fold after two rounds.

## Results

### Construction of a lentiviral vector encoding a tumor-specific CIR

**Figure 1:**
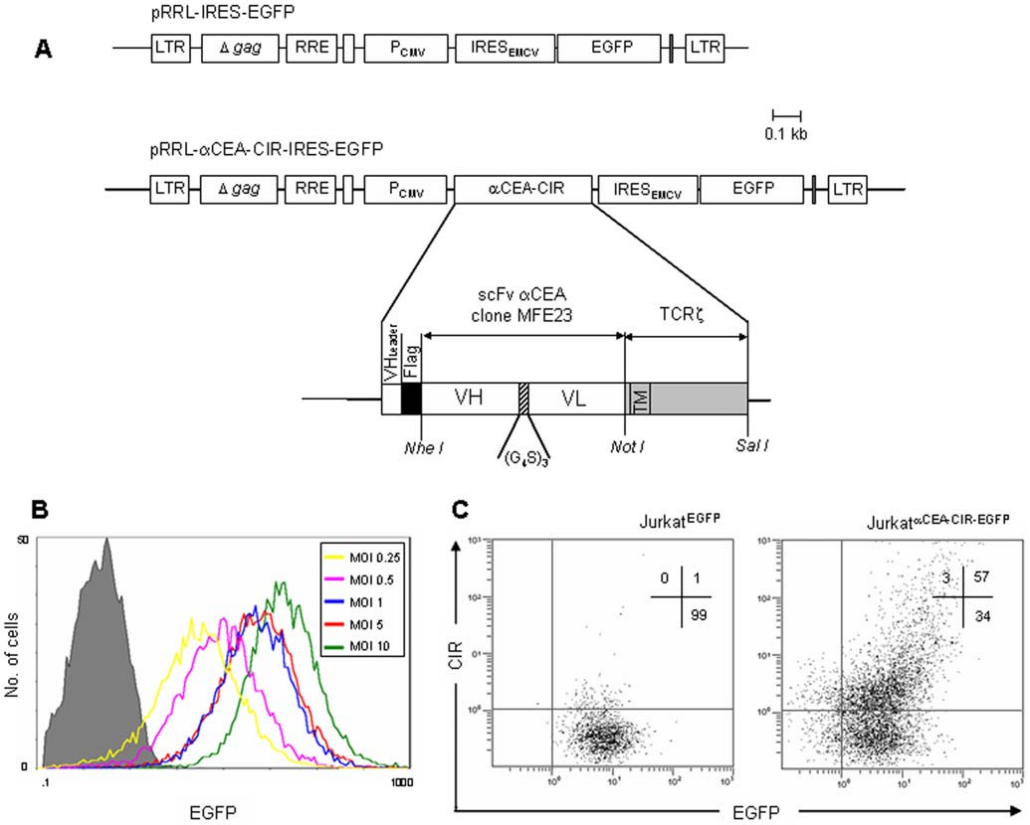
Schematic representation of lentiviral vector constructs. (A) Control monocistronic vector (pRRL-IRES-EGFP) containing only the enhanced-green fluorescent protein (EGFP) gene and bicistronic vector (pRRL-αCEA-CIR-IRES-EGFP) containing the chimeric immune receptor (CIR) gene and the EGFP sequence. LTR, long terminal repeats; ΔGAG, ATG-deleted group specific antigen; RRE, Rev-responsive *cis*-acting element; CMV promoter; ECMV IRES, enceohalomyocarditis virus internal ribosomal entry site. (B) FACS analysis of EGFP expression after transduction of Jurkat cells with EGFP encoding lentiviral vectors at different MOIs, ranging from 0.25 to 10. (C) FACS analysis of EGFP and cell surface CIR expression after transduction of Jurkat cells with CIR-EGFP encoding lentiviral vectors at MOI of 1.

In order to generate a model for validating a selection platform based on molecular and cellular events associated with T cell activation, we constructed the HIV-1 based lentiviral vector pRRL-αCEA-CIR-IRES-EGFP (Fig. 1A), containing a bicistronic expression cassette encoding a TCRζ-based CIR and EGFP, as reporter gene. The CIR comprises a human immunoglobulin signal peptide, a FLAG epitope, and the anti-carcinoembrionyc antigen (CEA) scFv antibody MFE23 [15], fused to the transmembrane and cytoplasmic regions of the human TCRζ-chain [16].

The human T cell line Jurkat was used as a model system to express the anti-CEA CIR and to test their ability to initiate T cell activation in response to tumor specific cell surface antigen. Jurkat cells were transduced with VSV-G pseudotyped lentiviral vectors encoding EGFP at different MOIs and flow cytometry analysis was used to evaluate the level of EGFP expression (Fig. 1B). Since transduction at low MOIs did not result in a substantial decrease of the EGFP expression, Jukat cells were infected at MOI 1 with VSV-G pseudotyped lentiviral vectors encoding both EGFP alone (EGFP) or anti-CEA CIR and EGFP (αCEA-CIR-EGFP), to ascertain expression of a single antibody species per infected cell. Flow cytometry analysis (Fig. 1C) revealed that in both cases over 95% of the cells were EGFP^+^. Analysis of cell-surface CIR expression demonstrated than approximately 60% of Jurkatα^CEA-CIR-EGFP^ cells expressed the FLAG-tagged anti-CEA CIR at detectable levels, although there was considerable heterogeneity in the absolute levels of expression (Fig. 1C). The difference between the absolute levels of EGFP and CIR expression may be due to the different characteristics and location of both proteins.

### Antigen-specific activation of T cells expressing tumor-specific CIRs

**Figure 2:**
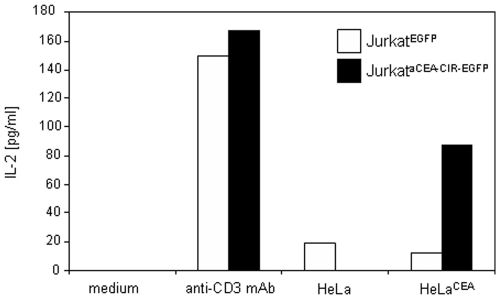
IL-2 production by Jurkat^EGFP^ and Jurkatα^CEA-CIR-EGFP^ cells stimulated either with plastic immobilized anti-CD3 mAb or target cells (E∶T = 1∶1; HeLa or HeLa^CEA^) for 48 hours.

**Figure 3:**
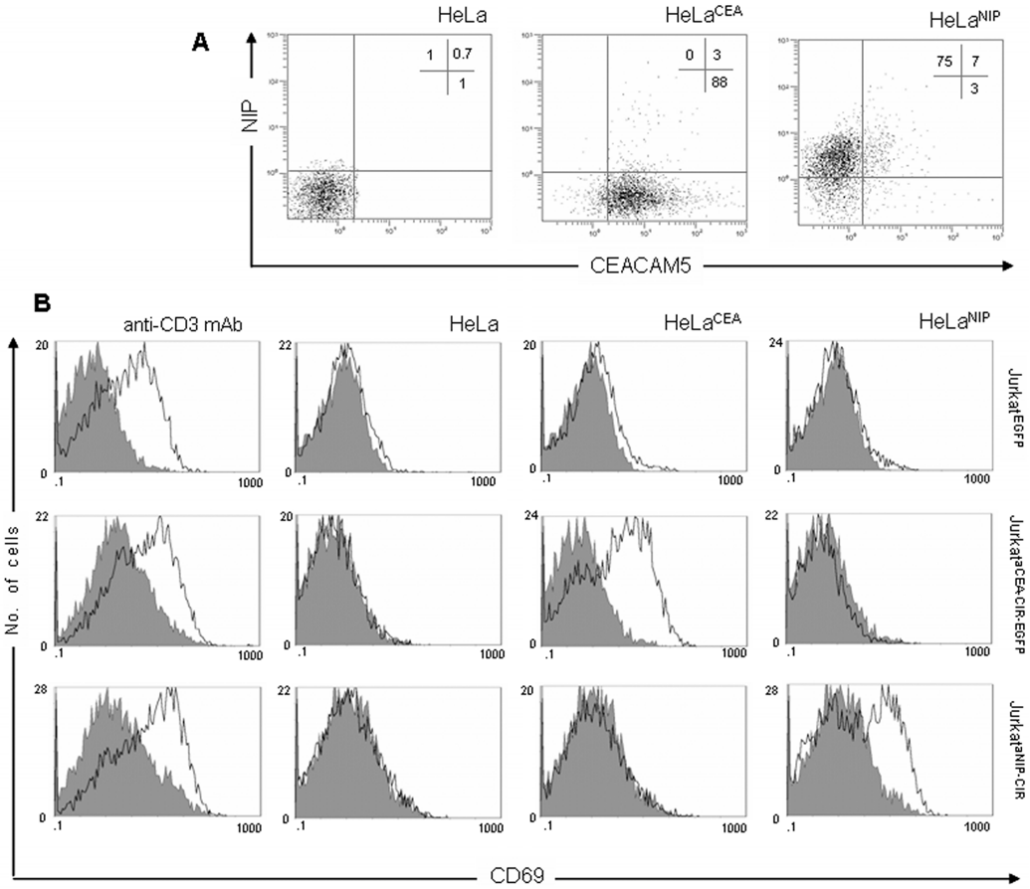
CIR-mediated activation of human T cells. (A) Cell-surface expression of CEACAM5 (CEA) and NIP-modified molecules on HeLa, HeLa^CEA^ and HeLa cells labeled with 2.5 µg/mL of the hapten (HeLa^NIP^). (B) FACS analysis of CD69 expression by Jurkat^EGFP^, Jurkatα^CEA-CIR-EGFP^ and Jurkatα^NIP-CIR^ stimulated either with immobilized anti-CD3 mAb or target cells (E∶T = 1∶1; HeLa, HeLa^CEA^ or HeLa^NIP^) for 16 hours.

We then examinated the activity of the FLAG-tagged anti-CEA CIR as a functional receptor molecule. Jurkatα^CEA-CIR-EGFP^ cells could be activated to secrete IL-2, upon antigen-mediated ligation of their binding domain by plastic immobilized CEA (iCEA) or membrane-bound CEA (mCEA) (Fig. 2). We next investigated the expression of the very early activation antigen CD69 in Jurkatα^CEA-CIR-EGFP^ and Jurkat^EGFP^ cells co-cultured overnight in the presence of CEA-negative (HeLa) or CEA-positive (HeLa^CEA^) cells (Fig. 3A). The results of a representative experiment are shown in Figure 3B. On average CD69^+^ cells accounted for 40±10% of Jurkatα^CEA-CIR-EGFP^ cells stimulated with CEA-positive cells. This result is similar to that observed after stimulation of Jurkatα^CEA-CIR-EGFP^ cells with plastic immobilized anti-CD3 mAb (Fig. 3B). In these conditions the peak expression of cell surface CD69 occurred between 12 and 24 h (data not shown). Stimulation of Jurkatα^CEA-CIR-EGFP^ and Jurkat^EGFP^ cells with plastic immobilized anti-CD3 mAb or stimulation of Jurkatα^CEA-CIR-EGFP^ cells with HeLa^CEA^ cells resulted in a marked increase in EGFP reporter expression as compared to unstimulated cells (data not shown).

We have demonstrated previously that an anti-NIP CIR is able to mediate specific recognition of its cognate antigen, either immobilized (NIP-BSA conjugates) or expressed on a cell surface, resulting in the production of IL-2 by transfected Jurkat T cells [16], [17]. The CIR comprises a human immunoglobulin signal peptide, the anti-NIP scFv antibody B1.8, fused to the transmembrane and cytoplasmic regions of the human TCRζ-chain [17]. In the current study, we found consistently that anti-NIP CIR-expressing Jurkat cells (Jurkatα^NIP-CIR^) could be specifically triggered, after incubation with NIP-modified target HeLa cells (HeLa^NIP^), to express CD69 (Fig. 3B). The level of CD69 expression was similar than that observed in the same cell population in response to plastic-immobilized anti-CD3 mAb (Fig. 3B). Importantly, T cell activation remained strictly antigen-specific with NIP-negative HeLa or HeLa^CEA^ cells unable to induce CD69 expression in Jurkatα^NIP-CIR^ cells (Fig. 3). Jurkat^EGFP^ cells or Jurkatα^NIP-CIR^ could not be stimulated by HeLa or HeLa^CEA^ cells (Fig. 3).

**Figure 4:**
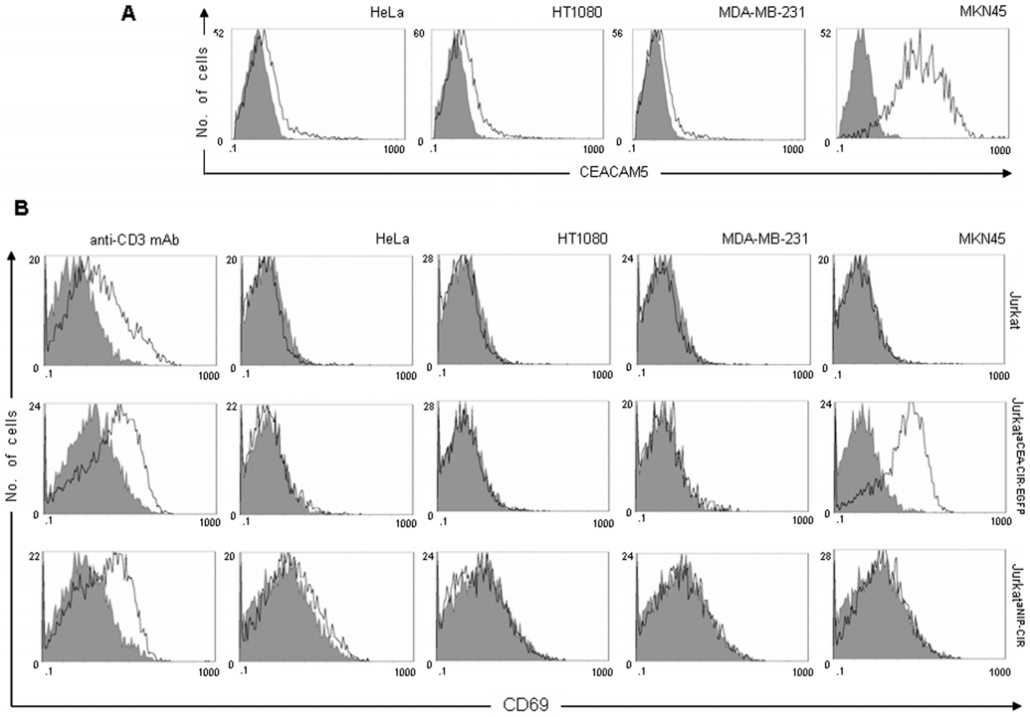
CIR-mediated activation of human T cells. (A) Cell-surface expression of CEACAM5 (CEA) on HeLa, HT1080, MDA-MB-231 and MKN45 cells. (B) FACS analysis of CD69 expression by Jurkat, Jurkatα^CEA-CIR-EGFP^ and Jurkatα^NIP-CIR^ stimulated either with immobilized anti-CD3 mAb or target cells (E∶T = 1∶1; HeLa, HT1080, MDA-MB-231 or MKN45) for 16 hours.

To further demonstrated the specificity of CIR-mediated activation, CIR-negative parental Jurkat cells or CIR-positive Jurkat cells (Jurkatα^CEA-CIR-EGFP^ or Jurkatα^NIP-CIR^) were co cultured with a panel of human unmodified tumor cell lines (Fig. 4). Only Jurkatα^CEA-CIR-EGFP^ cells expressed CD69 after co culturing with CEA-positive MKN45 cells (Fig. 4). Parental Jurkat cells or anti-NIP CIR-expressing Jurkat cells (Jurkatα^NIP-CIR^) could not be activated to express CD69 with any of the tumor cell lines tested (Fig. 4). Furthermore, the viability of Jurkat^EGFP^, Jurkatα^CEA-CIR-EGFP^ and Jurkatα^NIP-CIR^ cells was not affected when co-cultured for 16 hours in the presence of different types of target cells (data not shown).

### Antigen-specific activation of T cells expressing tumor-specific CIRs in the presence of competitor populations

**Figure 5:**
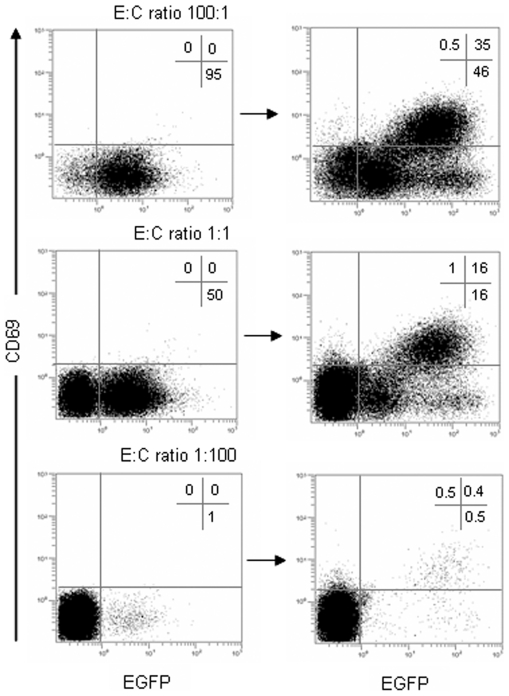
Effect of competitor populations on CIR-mediated T cell activation. CIR-positive Jurkatα^CEA-CIR-EGFP^ effector (E) cells were stimulated with CEA-positive target cells for 16 hours in the presence of increasing amounts of CIR-negative Jurkat competitor (C) cells. The expression of CD69 on E∶C ratios ranging from 100∶1 to 1∶100 was measured by FACS on pre-activation (left panels) and post-activation (right panels) mixtures.

We next studied the effect of competitor non-transduced Jurkat T cells on T cell activation mediated through the anti-CEA CIR. Jurkatα^CEA-CIR-EGFP^ cells were cultured on HeLa^CEA^ monolayers in the presence of increasing amounts of CIR-negative competitor Jurkat cells, and the expression of CD69 was analyzed by flow cytometry (Fig. 5). Our results indicate that in all these conditions the presence of competitor cells did not affect the CIR-mediated induction of CD69 expression (Fig. 5). At effector-to-competitor ratios ranging from 100∶1 to 1∶100 the percentage of the EGFP^+^ cells that expressed CD69 was kept close to 40% regardless of the number of competitor cells.

### Selection and enrichment of T cells expressing tumor-specific CIRs

**Figure 6:**
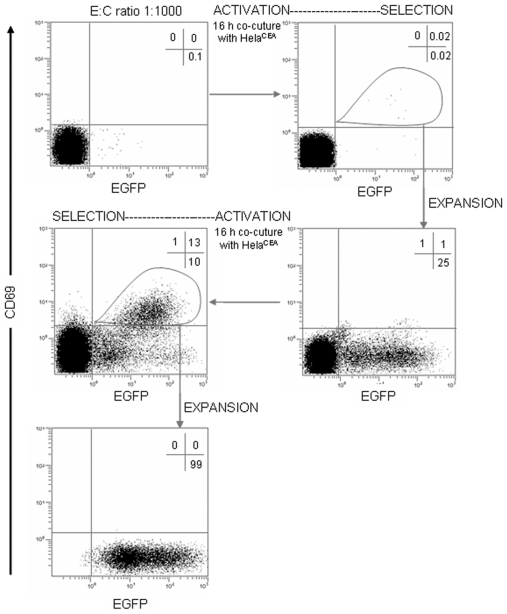
Selection of CIR-activated T cells. Jurkatα^CEA-CIR-EGFP^ effector (E) cells and CIR-negative Jurkat competitor (C) cells at a E∶C mixing ratio 1∶1000 were stimulated with CEA-positive target cells for 16 hours and further sorted on the basis of EGFP and CD69 expression. After a period of cell expansion the activation/selection cycle was repeated.

**Figure 7:**
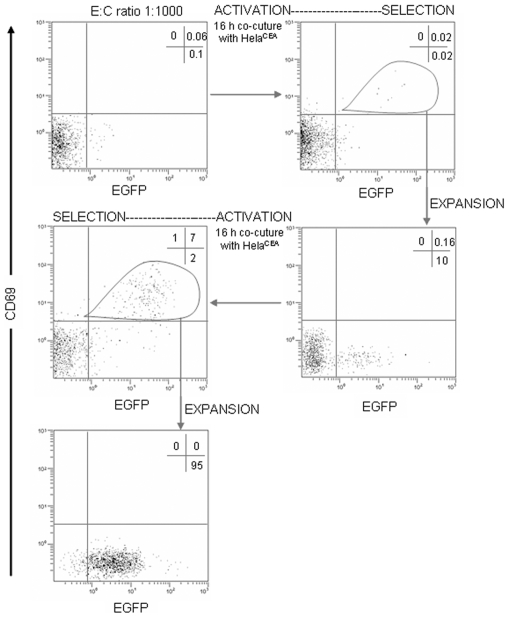
Selection of CIR-activated T cells. Jurkatα^CEA-CIR-EGFP^ effector (E) cells and CIR-positive Jurkatα^NIP-CIR^ competitor (C) cells at a E∶C mixing ratio 1∶1000 were stimulated with CEA-positive target cells for 16 hours and further sorted on the basis of EGFP and CD69 expression. After a period of cell expansion the activation/selection cycle was repeated.

To determine whether T cells expressing CIRs on their surfaces could serve as a platform for screening and isolating binders to tumor specific cell surface antigen, Jurkatα^CEA-CIR-EGFP^ cells and CIR-negative non-transduced Jurkat cells (Fig. 6) or CIR-positive transfected Jurkat cells (Jurkatα^NIP-CIR^) (Fig. 7) were mixed at decreasing concentrations of the former, with a total number of 3×10^7^ and incubated overnight with confluent monolayers of HeLa^CEA^ cells, and the sensitivity of the isolation and enrichment process evaluated. Mixed Jurkat T cells were recovered from the tumor cell monolayer by EDTA treatment, ficoll purified, washed twice with medium and incubated with anti-CD69 PE mAb. At a mixing ratio 1∶1000 a single round of selection by direct FACS sorting of EGFP^+^CD69^+^ cells, resulted in a two-log enrichment of anti-CEA CIR expressing Jurkatα^CEA-CIR-EGFP^ cells, from the background CIR-negative Jurkat cell population (Fig. 6) or CIR-positive Jurkatα^NIP-CIR^ cells. The sorted Jurkatα^CEA-CIR-EGFP^/1S population was propagated and submitted for an additional round of activation/selection on HeLa^CEA^ cell monolayers. After staining with anti-CD69 PE mAb and FACS sorting nearly 100% of the cells expressed EGFP (Jurkatα^CEA-CIR-EGFP^/2S). Overall, CIR-mediated activation of T cells combined with FACS sorting of CD69-expressing activated T cells resulted in a 10^3^ fold-enrichment in a tandem two-step round of selection.

### Functional and phenotypic characterization of selected T cells expressing tumor-specific CIRs

**Figure 8:**
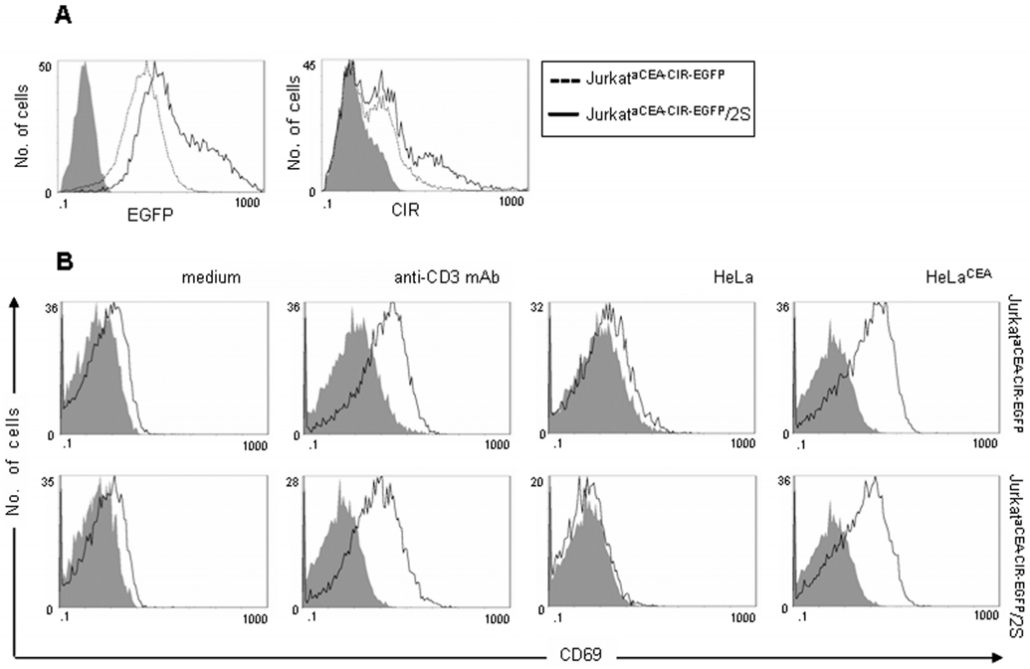
Phenotypic and functional characterization of selected Jurkatα^CEA-CIR-EGFP^ cells. (A) Comparative analysis of EGFP and cell surface CIR expression of the initial (Jurkatα^CEA-CIR-EGFP^) and the selected (Jurkatα^CEA-CIR-EGFP^/2S) cell population after two rounds of activation/selection on CEA-positive target cells. (B) Comparative analysis of CD69 expression by Jurkatα^CEA-CIR-EGFP^ and Jurkatα^CEA-CIR-EGFP^/2S cell populations after stimulation either with immobilized anti-CD3 mAb or target cells (E∶T = 1∶1; HeLa or HeLa^CEA^).

After the second round of activation/selection, the selected population (Jurkatα^CEA-CIR-EGFP^/2S) was expanded and characterized phenotypic and functionally. Jurkatα^CEA-CIR-EGFP^/2S cells showed higher levels of EGFP and cell surface FLAG-tagged anti-CEA CIRs (with MFI values enhanced ∼2-fold) than the initial Jurkatα^CEA-CIR-EGFP^ cells (Fig. 8A). Jurkatα^CEA-CIR-EGFP^/2S cells could be activated to express CD69 or to produce IL-2, upon interaction with membrane-bound CEA (mCEA) or immobilized anti-CD3 mAb (Fig. 8B). Sequence analysis of antibody variable domains of 20 individual clones revealed no differences between the initial Jurkatα^CEA-CIR-EGFP^ cells and the selected Jurkat Jurkatα^CEA-CIR-EGFP^/2S cells with the exception of punctual one-nucleotide changes, derived from the PCR amplification (data not shown).

## Discussion

In this study, we describe the design and use of a mammalian display platform to select scFv antibodies on complex mixtures of antigens expressed on cell surfaces. A limiting step in the selection by cell panning of phage antibodies is the high background binding of non-specific phages [4]. To overcome this limitation, we designed a positive selection method based on the display of scFv antibodies on the surface of T lymphocytes, in the context of activating molecules. The use of a chimeric scFv-TCRζ receptor as a display fusion partner may result in gene expression in successful cases of interaction.

In fact, we demonstrated that upon encountering specific antigen on tumor cell surface, human T cells harboring anti-tumor scFv-TCRζ receptors are able to undergo specific activation, including up-regulation of the early T-cell activation marker CD69. Combining CIR-mediated activation with FACS sorting of CD69^+^ T cells it is possible to detect and purify tumor-specific T cells with an enrichment factor of at least 10^3^-fold after two rounds. However, the strength of this lymphocyte display platform does not rely only on the enrichment factor, but in rescuing functional scFv antibodies capable of forming a functional immune synapse.

Recent studies have established that multidomain constructs comprising the signaling region of CD28 in series with the signaling domain of the TCRζ chain (dual scFv-CD28-TCRζ chimeric receptor) mediated enhanced cytokine production, and preserved cell viability [18]. Dual CIR with optimized spacer sequences, to provide accessibility and extended orientation, are ideal for surface display of antibody libraries in T lymphocytes.

In addition to the advantages of all mammalian expression libraries this novel positive selection T cell–surface display system avoids the use of elution procedures so that high affinity antibodies cannot get lost during selection. Furthermore, the interface between the tumor cell and the CIR-expressing T cell might allow selecting human antibodies towards their cognate antigens when present in their natural setup. Antibodies against transmembrane cell surface proteins that are difficult to purify in a native conformation and against epitopes that are not visible with the current display technologies might benefit from this platform. The use of lentiviral-based vectors allows to precisely dose infection rates, ensuring expression of no more than one antibody per cell. However, one major drawback of antibody screening in mammalian cells is the relative small number of cells that can be handled at time. Thus, whereas phage display routinely allows for the screening of 10^12^ clones in a single panning round [2], the throughput of a mammalian screening procedure in an one-antibody-per-cell format is in the range of 10^6^ to 10^7^ clones that can be analyzed concomitantly [8].

This “repertoire diversity” limitation might be ameliorated thanks to its better fit to specific scenarios, for example *in vivo* selections. Systemically administered bacteriophages have to survive destruction by immune effector mechanisms [19]―which counts for a fast depletion *in vivo―*, avoid non-specific adhesion to vessel walls [20] and avoid sequestration by organ systems such as the Kuppfer cells [21], thus, diminishing the actual repertoire size at the “point of selection” (whether a solid tumor, a disease model, organ-specific vasculature, etc). In addition phage particles represent an inert carrier, with void or limited extravasation ability, which allows the recovery of displayed peptides homing preferentially to the target tissue due to their binding to tissue-specific vascular receptors [22], but poses further restrictions for the *in vivo* selections beyond the luminal surface of the vasculature. The present platform overcomes these limitations: unlike viral particles, immune effector cells can migrate actively and efficiently through microvascular walls, and penetrating the core of solid tumors. In this context, a repertoire of systemically administered CIR-display T lymphocytes can move through the circulatory and lymphatic systems and extravasate actively towards the site of tissue damage. We could select tumor-specific T cells as consequence of a process of extravasation, mediated by cellular adhesion molecules, and of a process of retention and activation, mediated by the CIR.

## Materials and Methods

### Cells and culture conditions

293T cells (human embryo kidney epithelia; CRL-11268), HeLa cells (human cervix carcinoma; CCL-2), HT-1080 cells (human fibrosarcoma; CCL-121), MKN45 cells (human stomach adenocarcinoma; JCRB-0254), and MDA-MB231 cells (human breast adenocarcinoma; HTB-26) were grown in DMEM supplemented with 10% (vol/vol) heat-inactivated FCS, (Invitrogen Life Technologies, Gaithersburg, MD, USA), referred as to DMEM complete medium (DCM). Jurkat clone E6-1 (TIB-152) cells were maintained in RPMI-1640 (Invitrogen Life Technologies) supplemented with heat inactivated 10% FCS, referred as to RPMI complete medium (RCM). All of these cell lines were obtained from the American Type Culture Collection (Rockville, MD, USA). HeLa^CEA^ cells [23] were grown in RCM supplemented with 750 µg/ml G418 (Invitrogen Life Technologies). All cell lines were routinely screened for the absence of mycoplasm contamination by PCR using the Mycoplasm Plus TM Primer Set (Stratagene, Cedar Creek, TX, USA).

### Hapten modification of target cells

HeLa cells were extensively washed in PBS, counted, and adjusted to a concentration of 10^7^/mL. A fresh 50 mg/ml solution of NIP-CAP-Osu (succinimide ester of 3-nitro-4-hydroxy-5-iodophenylacetic acid spaced with caproic acid) (Sigma Biosciences, St. Louis MO, USA) in dry dimethylformamide was added to the cell suspension to give a final concentration of 2.5 µg/mL. The cells were incubated for 1 hour at 37°C and then washed three times with 15 mL of cold PBS supplemented with 10% FCS.

### Antibodies and reagents

The monoclonal antibodies (mAbs) used included: M2 (anti-FLAG, Sigma Biosciences); 9E10 (anti-c-myc, Abcam, Cambridge, UK), C6G9 (anti-human CD66e/CEA, Sigma Biosciences); BD1690 (anti-HIV p24, Abcam); SPVT3b (anti-human CD3, Zymed, San Francisco, CA, USA) and BG5 (biotinylated anti-human IL-2, Endogen, Woburn, MA, USA). For direct staining, the PE-conjugated FN50 mAb (anti-human CD69, BD Biosciences, San José, CA, USA) was used. The polyclonal antibodies used included: rabbit anti-human IL2 (Endogen), horseradish peroxidase (HRP)-conjugated goat anti-mouse IgG (Fc specific; Sigma Biosciences); PE-conjugated goat F(ab')_2_ fragment anti-mouse IgG (Fcγ Fragment Specific, Jackson Immuno Research, Newmarket, UK); and biotinylated goat anti-HIV p24 (Abcam). Streptavidin-HRP polymer, human IL-2, and human CEA were from Sigma Biosciences and HIV-p24 core protein was from Jena Bioscience GmbH (Jena, Germany).

### Vector construction and preparation of lentiviral vector stocks

The HIV-derived four-plasmid system was kindly provided by D. Trono (Department of Microbiology and Molecular Medicine, University of Geneva, Switzerland). The transfer vector pRRL-IRES-EGFP contains a cytomegalovirus (CMV) promoter that drives an enhanced-green fluorescent protein (EGFP) expression cassette [24]. To construct the vector pRRL-FLAGαCEA-TCRζ-IRES-EGFP, the plasmid pVAC.αNIP-TCRζ was digested with NotI and XbaI to remove a PstI site and religated to form pVAC.αNIP-TCRζ-Not/Xba in which an oligonucleotide carrying the FLAG sequence (Table 1, oligonucleotides 1 and 2) was introduced between XmaI and PstI to generate the plasmid pVAC.FLAGαNIP-TCRζ-Not/Xba. The coding sequence was restored by inserting the HindIII/BstEII fragment from plasmid pVAC.FLAGαNIP-TCRζ-Not/Xba into the HindIII/BstEII digested backbone of plasmid pVAC.αNIP.TCRζ rendering the plasmid pVAC.FLAGαNIP-TCRζ. The MFE-23 scFv gene (αCEA) was PCR amplified from pVAC.αCEA-TCRζ with primers 3 and 4 (Table 1) and the sequence was verified using primers 5 and 6. The NheI/NotI-cleaved PCR fragment was ligated into the NheI/NotI digested backbone of plasmid pVAC.FLAGαNIP-TCRζ to obtain the plasmid pVAC.FLAGαCEA-TCRζ. To aid cloning, a NotI site was removed from pRRL-IRES-EGFP by digestion using NotI, blunting and religation (pRRL-IRES-EGFP^NotKO^). The FLAGαCEA-TCRζ coding sequence was PCR amplified from plasmid pVAC.FLAGαCEA-TCRζ with primers 7 and 8 (Table 1), digested with BglII and SalI and subcloned into the BamHI/XhoI-digested backbone of the plasmid pRRL-IRES-EGFP^NotKO^, obtaining the vector pRRL.FLAGαCEA-TCRζ-IRES-EGFP (called from now pRRL-CIR-IRES-EGFP). Lentiviral particles (ACEA-CIR-EGFP and EGFP) were produced by cotransfection of 293T cells by calcium phosphate precipitation as described previously [24].

**Table 1:** Oligonucleotide sequences^a^.

Name	Sequence (5′-3′)
**1**	CCGGGGGCCCACTCCGACTACAAAGACGATGACGACAAGGCTAGCCAGGTGCAGCTGCA
**2**	GCTGCACCTGGCTAGCCTTGTCGTCATCGTCTTTGTAGTCGGAGTGGGCCC
**3**	GCTAGCCAGGTGCAGCTGCAGCAGTCT
**4**	CTCTGTAGCGGCCGCCCGTTT
**5**	CAGGAAACAGCTATGAC
**6**	TAAAACGACGGCCAG
**7**	ACAGATCTATGGACTGGACCTGGAGGGT
**8**	AAATGTCGACTTATTAGCGACGAGGGGGCAG

a Sequences of the various oligonucleotides applied for the construction of the vectors, and subsequent verification of vector sequences.

### Determination of vector titers

An ELISA directed against the p24 capsid protein was performed for tittering the physical particles (PP). PP number was in the range of 1×10^7^–1×10^8^ PP/ml in both lentiviral vector preparations (ACEA-CIR-EGFP and EGFP). Biological titration was performed as previously described [16]. The infective particles (transducing units, TU/ml) were 4×10^7^ TU/ml for the lentivirus ACEA-CIR-EGFP and 2×10^7^ TU/ml for the lentivirus EGFP.

### Genetic modification of human T cells

Exponentially growing Jurkat cells (1×10^5^) were incubated overnight in 96-well plates with lentiviral stocks (αCEA-CIR-EGFP or EGFP) at different multiplicities of infection (MOI), ranging from 0.25 to 10 in a final volume of 200 ml of their appropriate medium. The cells were washed and further cultured for 48 h. Jurkat cells were transfected with the expression vector pCEP4/αNIPCD3ζ [16], using Superfect (Qiagen GmbH, Hilden, Germany) and selected in RCM with 400 µg/ml hygromycin B (Invitrogen Life Technologies). Cells were analyzed for expression of EGFP and CIR (αCEA or αNIP) by FACS.

### Flow cytometry

For phenotypic analysis cells were incubated with appropriate dilutions of PE-conjugated mAbs or PE-conjugated isotype negative control for 30 min at 4°C. The expression of CEACAM5 (CEA) on HeLa, HeLa^CEA^, HT1080, MDA-MB-231 and MKN45 cells was studied as previously described [25]. Cell-surface expression of amino-terminal FLAG-tagged CIR (αCEA-CIR) was evaluated after incubation of cells with anti-FLAG mAb followed by incubation with PE-conjugated goat F(ab')_2_ anti-mouse IgG. Cell-surface expression of α-NIP-CIR was evaluated using a FITC-conjugated goat anti-serum to mouse λ-chain (Southern Biotechnology Associates, Birmingham, AL; USA). The relative amount of NIP bound to hapten-modified HeLa cells (HeLa^NIP^) was estimated by indirect immunofluorescence using saturating amounts of a purified B1.8 scFv antibody fragment (LAV-17), anti-c-myc mAb and a PE-conjugated goat F(ab')_2_ fragment anti-mouse IgG. All samples were analyzed using an EPICS XL flow cytometer (Beckman Coulter).

### T-cell activation and selection

Non-transduced Jurkat, Jurkatα^CEA-CIR-EGFP^ or Jurkat^EGFP^ cells (10^5^/well) were incubated with plastic immobilized CEA (1 µg/well) or anti-CD3 mAb (1 µg/well) in round-bottom 96-well plates (BD Biosciences). For small-scale co-culture assays, CIR-positive (Jurkatα^CEA-CIR-EGFP^ or Jurkatα^NIP-CIR^), CIR-negative (Jurkat or Jurkat^EGFP^), or mixtures of both effector T cells (5×10^4^/well) were incubated with different types of target cells, at various effector to target ratios. The plates were incubated at 37°C in 5% CO_2_. After 16 h or 40 h, cells were collected and the surface expression of CD69 examined by FACS analysis. Supernatants were harvested and assayed for IL-2 activity by ELISA.

For medium-scale co-cultures, CIR-positive (Jurkatα^CEA-CIR-EGFP^ or Jurkatα^NIP-CIR^), CIR-negative (Jurkat), or mixtures of both effector (Jurkatα^CEA-CIR-EGFP^:Jurkat or Jurkatα^CEA-CIR-EGFP^:Jurkatα^NIP-CIR^), T cells (3×10^7^) were incubated on HeLa or HeLa^CEA^ monolayers at a 1∶1 ratio. After 16 h, cells were harvested with EDTA (Sigma Biosciences), washed, and then Jurkat cells isolated by density-gradient centrifugation. Viable cell counts were determined by trypan blue exclusion. The resultant cells were stained with PE-conjugated anti-CD69 mAb and EGFP^+^CD69^+^ populations further isolated by FACS using a FACSVantage cell sorter (BD Biosciences)

### Molecular characterization

Total RNA was extracted from unselected and selected Jurkatα^CEA-CIR-EGFP^ cells with the RNeasy Plus Mini Kit (Qiagen). cDNA synthesis was performed with cDNA Synthesis Kit for RT-PCR (AMV) (Roche Diagnostics GmbH, Mannheim, Germany). The anti-CEA scFv was PCR amplified with primers 4 and 7, and sequenced with primers 5 and 6 (Table 1).
